# Supplementary material for: Robust weak antilocalization due to spin-orbital entanglement in Dirac material Sr3SnO
Source: Nat Commun. 2020 Mar 3;11:1161. doi: 10.1038/s41467-020-14900-1 (PMC7054336; doi:10.1038/s41467-020-14900-1)
Supplement: Supplementary file 1 — Supplementary Information [file 41467_2020_14900_MOESM1_ESM.pdf]

Supplementary Information for "Robust weak antilocalization due to spin-orbital entanglement in Dirac material Sr_3SnO"

H. Nakamura,[1,*] D. Huang,[1] J. Merz,[1] E. Khalaf,[1,2] P. Ostrovsky,[1,3] A. Yaresko,[1] D. Samal,[4,5] and H. Takagi[1,6,7]

[1]*Max Planck Institute for Solid State Research, 70569 Stuttgart, Germany*
[2]*Department of Physics, Harvard University, Cambridge MA 02138, USA*
[3]*L. D. Landau Institute for Theoretical Physics RAS, 119334 Moscow, Russia*
[4]*Institute of Physics, Bhubaneswar 751005, India*
[5]*Homi Bhabha National Institute, Mumbai 400085, India*
[6]*Department of Physics, University of Tokyo, 113-0033 Tokyo, Japan*
[7]*Institute for Functional Matter and Quantum Technologies, University of Stuttgart, 70569 Stuttgart, Germany*

* hnakamur@uark.edu; present address: Department of Physics, University of Arkansas, AR 72701, USA

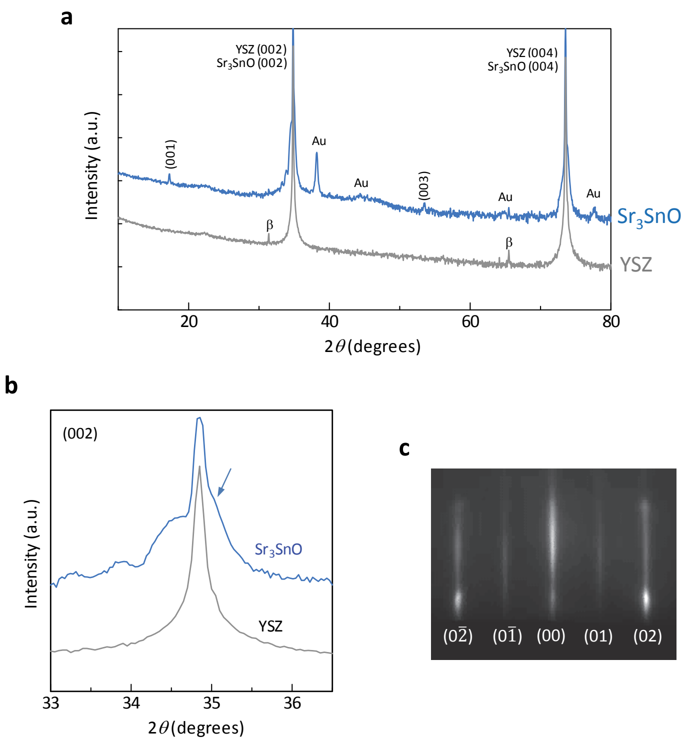

Supplementary Figure 1. **Characterization of antiperovskite films.** **a** X-ray $2\theta - \theta$ scan of Sr_3SnO. For a comparison, the XRD scan of bare YSZ substrate is also shown. The Miller indices correspond to those of the film unless otherwise noted. Data are shifted vertically for clarity. β denotes the diffraction peak related to Cu K_β line which is not filtered perfectly. **b** Magnified $2\theta - \theta$ scan around Sr_3SnO (002) diffraction, where the diffraction from the film is marked with the arrow. The weak oscillation in lower angle comes from a SrO buffer layer. Data are shifted vertically for clarity. **c** The RHEED image taken along [100] direction of the YSZ substrate after the growth of 100 nm Sr_3SnO film.

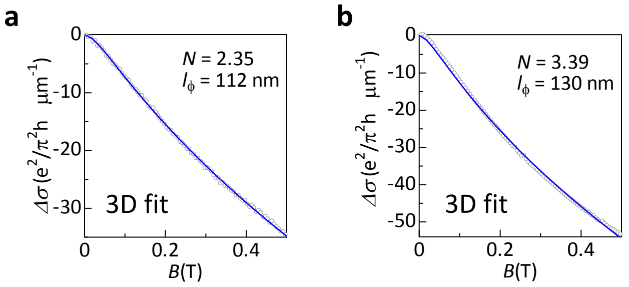

Supplementary Figure 2. **Three-dimensional WAL analysis for quasi-2D films.** Low-field MC for quasi-2D films at **a** low ($n = 1.8 \times 10^{19}\text{cm}^{-3}$) and **b** high ($n = 7.2 \times 10^{19}\text{cm}^{-3}$) hole density. Experimental data are shown in open circles. Theoretical fits based on 3D formula are shown in solid lines.

Supplementary Table 1. The WAL fitting results based on 3D and 2D formulae for 3D films

Sample No.	T_{min}[a] (K)	n_p ($\times 10^{19}$ cm^{-3})	d (nm)	N_{3Dfit}	$l_{\phi,\mathrm{3Dfit}}$ (nm)	N_{2Dfit}	$l_{\phi,\mathrm{2Dfit}}$ (nm)
1	2	1.70	100	1.40 ± 0.22	65 ± 1.3	1.59 ± 0.1	47 ± 1.2
2	0.42	1.80	200	1.28 ± 0.25	321 ± 37	1.21 ± 0.14	159 ± 8.8
3	1.8	2.11	90	0.89 ± 0.11	313 ± 15	0.69 ± 0.03	98.5 ± 3.1
4	4	2.20	300	0.84 ± 0.3	364 ± 89	1.16 ± 0.4	148 ± 18
5	3	2.20	150	1.17 ± 0.19	246 ± 41	1.23 ± 0.14	110 ± 6.6
6	4.5	2.30	300	0.55 ± 0.40	283 ± 100	1.00 ± 0.7	146 ± 29
7	2	4.92	105	2.56 ± 0.37	174 ± 13	1.31 ± 0.14	118 ± 4.6
8	2	6.09	160	2.60 ± 0.6	147 ± 52	3.74 ± 1.6	84.4 ± 22
9	3	9.00	100	1.95 ± 0.26	134 ± 15	1.87 ± 0.1	72.8 ± 2.5
10	2	11.1	160	2.46 ± 0.1	162 ± 8.1	3.2 ± 0.2	87.0 ± 2.7
11	4.5	13.0	100	2.48 ± 0.45	107 ± 14	2.6 ± 0.33	64.0 ± 4.0

[a] the lowest temperature at which $\Delta\sigma$ for fitting was measured

Supplementary Table 2. The WAL fitting results based on 3D and 2D formulae for quasi-2D films

Sample No.	$T_{\rm min}$[a] (K)	n_p ($\times 10^{19}$ cm^{-3})	d (nm)	$N_{\rm 3Dfit}$	$l_{\phi,\rm 3Dfit}$ (nm)	$N_{\rm 2Dfit}$	$l_{\phi,\rm 2Dfit}$ (nm)
12	0.45	1.75	50	2.35 ± 0.1	112 ± 5	1.02 ± 0.04	71.4 ± 1.3
13	0.42	7.20	50	3.39 ± 0.25	127 ± 11	1.46 ± 0.01	76.5 ± 0.3

[a] the lowest temperature at which $\Delta\sigma$ for fitting was measured

Supplementary Note 1. Derivation of 3D weak (anti)localization formula

In order to describe the crossover between positive and negative magentoresistance in 3D we consider a generic model of a disordered metal with three types of impurities: potential, spin-orbit, and magnetic. This model is completely analogous to that used by Hikami, Larkin, and Nagaoka [1] for the 2D case. Magnetic impurities are included for generality and will be neglected in the end of the calculation. Following Supplementary Reference 1, we assume that impurity scattering amplitude has the form

$$f_{\alpha\beta}\left(\mathbf{n},\mathbf{n}'\right) = a\delta_{\alpha\beta} + ib\left(\mathbf{n}\times\mathbf{n}'\right)\sigma_{\alpha\beta} + \mathbf{s}\sigma_{\alpha\beta}. \quad (1)$$

The random scalar parameters a, b and the vector $\mathbf{s}$ obey a Gaussian distribution with zero average and

$$\left\langle a^2\right\rangle = \frac{1}{2\pi\nu\tau_0}, \quad \left\langle b^2\right\rangle = \frac{9}{4\pi\nu\tau_{\mathrm{SO}}}, \quad \left\langle \mathbf{s}_\alpha\mathbf{s}_\beta\right\rangle = \frac{\delta_{\alpha\beta}}{2\pi\nu\tau_m}.$$

This defines scattering times τ_0, τ_{SO}, and τ_m. Overall scattering rate is given by the imaginary part of the self energy or, equivalently, by the Fermi golden rule and equals

$$\frac{1}{\tau} = 2\pi\nu\langle f_{\alpha\beta}\left(\mathbf{n},\mathbf{n}'\right)f_{\beta\gamma}\left(\mathbf{n}',\mathbf{n}\right)\rangle_{\mathbf{n}'} = \frac{1}{\tau_0} + \frac{3}{\tau_{\mathrm{SO}}} + \frac{3}{\tau_m}.$$

Weak (anti)localization effect is due to interference between self-intersecting trajectories that differ by time reversal. Such pairs of trajectories are described by the Cooperon propagator that involves the following impurity vertex:

$$\begin{aligned}\Gamma &= \langle f\left(\mathbf{n},\mathbf{n}'\right)\otimes f\left(-\mathbf{n},-\mathbf{n}'\right)\rangle_{\mathbf{n},\mathbf{n}'} \\ &= \frac{1}{2\pi\nu\tau_0} - \frac{\boldsymbol{\sigma}\otimes\boldsymbol{\sigma}}{2\pi\nu\tau_{\mathrm{SO}}} + \frac{\boldsymbol{\sigma}\otimes\boldsymbol{\sigma}}{2\pi\nu\tau_{\mathrm{m}}}.\end{aligned} \quad (2)$$

For brevity, we write simply 1 instead of $1\otimes 1$. The averaging over both $\mathbf{n}$ and $\mathbf{n}'$ is justified provided the spin-orbit scattering is a relatively rare event and the electron velocity is fully randomized by potential scattering between two consecutive spin-orbit impurities.

It is convenient to introduce the following two combinations:

$$\mathbb{S} = \frac{1-\boldsymbol{\sigma}\otimes\boldsymbol{\sigma}}{4}, \qquad \mathbb{T} = \frac{3+\boldsymbol{\sigma}\otimes\boldsymbol{\sigma}}{4}. \quad (3)$$

These operators obey $\mathbb{S}^2 = \mathbb{S}$ and $\mathbb{T}^2 = \mathbb{T}$ and project onto the subspaces with total spin zero and one respectively. These subspaces are naturally called the singlet and triplet channels. The vertex Γ can be rewritten in this basis as

$$\Gamma = \frac{\mathbb{S}}{2\pi\nu}\left(\frac{1}{\tau} - \frac{6}{\tau_m}\right) + \frac{\mathbb{T}}{2\pi\nu}\left(\frac{1}{\tau} - \frac{4}{\tau_{\mathrm{SO}}} - \frac{2}{\tau_m}\right). \quad (4)$$

Here the spin-orbit and magnetic rates τ_{SO}^{-1} and τ_m^{-1} are regarded as small corrections to τ^{-1}; the latter is dominated by the potential scattering.

In the Cooperon ladder, the vertices Γ are connected by the pairs of Green functions

$$\begin{aligned}\Pi &= \int \frac{d\mathbf{p}}{(2\pi)^3} G^R\left(\mathbf{p}+\mathbf{q}\right)\otimes G^A\left(-\mathbf{p}\right) \\ &= 2\pi\nu\tau\left(1 - \tau Dq^2 - \frac{\tau}{\tau_\phi}\right),\end{aligned} \quad (5)$$

where we have included a phenomenological dephasing rate τ_ϕ^{-1}. Summation of the ladder diagrams leads to the following result:

$$\begin{aligned}C(q) = \Gamma\left(1-\Pi\Gamma\right)^{-1} &= \frac{\mathbb{S}}{2\pi\nu\tau^2}\left(Dq^2 + \frac{6}{\tau_m} + \frac{1}{\tau_\phi}\right)^{-1} \\ &+ \frac{\mathbb{T}}{2\pi\nu\tau^2}\left(Dq^2 + \frac{4}{\tau_{\mathrm{SO}}} + \frac{2}{\tau_m} + \frac{1}{\tau_\phi}\right)^{-1}.\end{aligned} \quad (6)$$

The small symmetry-breaking rates τ_{SO}^{-1} and τ_m^{-1} are kept only in the denominators. The weak (anti)localization correction is given by the integral of the Cooperon loop

$$\begin{aligned}\Delta\sigma &= -\frac{2e^2\nu D\tau^2}{\hbar}\int d\mathbf{q}\,\mathrm{Tr}\,C(q) \\ &= \frac{e^2}{\pi\hbar}\int\frac{d\mathbf{q}}{(2\pi)^3}\left[\left(q^2 + 6l_m^{-2} + l_\phi^{-2}\right)^{-1}\right. \\ &\qquad \left. -3\left(q^2 + 4l_{\mathrm{SO}}^{-2} + 2l_m^{-2} + l_\phi^{-2}\right)^{-1}\right].\end{aligned} \quad (7)$$

Here we have used the values $\mathrm{Tr}\,\mathbb{S} = -1$ and $\mathrm{Tr}\,\mathbb{T} = 3$ and introduced the scattering lengths $l_i = \sqrt{D\tau_i}$ corresponding to different types of impurities.

To include an external magnetic field, we replace $\mathbf{q}\mapsto\mathbf{q}+(2e/c)\mathbf{A}$. Hence the Cooperon dynamics is quantized in the transverse direction giving the sum over effective Landau levels

$$\begin{aligned}&\int\frac{d\mathbf{q}}{(2\pi)^3}\frac{1}{q^2+l_i^{-2}} \\ &\mapsto \frac{1}{4\pi l_B^2}\sum_n\int\frac{dq_z}{2\pi}\frac{1}{q_z^2 + l_B^{-2}(n+1/2) + l_i^{-2}}.\end{aligned} \quad (8)$$

In this expression, l_i^{-2} denotes one of the relevant mass terms from Supplementary Equation 7 and the magnetic length is $l_B = \sqrt{\hbar/4eB}$.

The sum and integral in Supplementary Equation 8 diverge in the ultraviolet limit. They should be cut at the ballistic scale when the Cooperon denominator is comparable to l^{-2}. This can be achieved in several ways. One possibility is to subtract a similar integral at zero magnetic field as was done in Supplementary Reference [2]. Alternatively, we can introduce a regulating term in the denominator in the following way:

$$\sum_n \int \frac{dq_z}{q_z^2 + l_B^{-2}(n+1/2) + l_i^{-2}} \mapsto \sum_n \int \frac{dq_z}{q_z^2 + l_B^{-2}(n+1/2) + l_i^{-2} + l^2\left[q_z^2 + l_B^{-2}(n+1/2) + l_i^{-2}\right]^2}$$

$$= \pi l_B \sum_n \left[\frac{1}{\sqrt{n+1/2+l_B^2/l_i^2}} - \frac{1}{\sqrt{n+1/2+l_B^2/l^2+l_B^2/l_i^2}}\right] = \pi l_B \left[\zeta\left(\frac{1}{2}, \frac{1}{2} + \frac{l_B^2}{l_i^2}\right) - \zeta\left(\frac{1}{2}, \frac{1}{2} + \frac{l_B^2}{l^2} + \frac{l_B^2}{l_i^2}\right)\right]. \quad (9)$$

Here ζ is the Hurwitz zeta function. In the argument of the second zeta function, l_i^{-2} can be neglected in favor of l^{-2}. Applying Supplementary Equations 8 and 9 to the weak localization correction Supplementary Equation 7, we obtain a general expression for the magnetoconductivity

$$\Delta\sigma(B) = \frac{e^2}{4\pi h l_B}\left[2\zeta\left(\frac{1}{2}, \frac{1}{2} + \frac{l_B^2}{l^2}\right) + \zeta\left(\frac{1}{2}, \frac{1}{2} + 6\frac{l_B^2}{l_m^2} + \frac{l_B^2}{l_\phi^2}\right) - 3\zeta\left(\frac{1}{2}, \frac{1}{2} + 4\frac{l_B^2}{l_{SO}^2} + 2\frac{l_B^2}{l_m^2} + \frac{l_B^2}{l_\phi^2}\right)\right]. \quad (10)$$

This 3D result is fully analogous to the corresponding 2D result of Hikami, Larkin, and Nagaoka [1] up to the replacement of digamma functions with Hurwitz functions.

For the description of experimental magnetoresistance, we assume $l_m^{-1} = 0$ since, qualitatively, magnetic scattering has a similar effect to dephasing. This yields

$$\Delta\sigma(B) = \frac{e^2}{4\pi h l_B}\left[2\zeta\left(\frac{1}{2}, \frac{1}{2} + \frac{l_B^2}{l^2}\right) + \zeta\left(\frac{1}{2}, \frac{1}{2} + \frac{l_B^2}{l_\phi^2}\right) - 3\zeta\left(\frac{1}{2}, \frac{1}{2} + 4\frac{l_B^2}{l_{SO}^2} + \frac{l_B^2}{l_\phi^2}\right)\right]. \quad (11)$$

This result describes the crossover from negative to positive magnetoconductivity with increasing magnetic field. In Eq. 1 of the main text, the number of independent channels N has been included into Supplementary Equation 11.

The Hurwitz zeta function has the following two asymptotic forms:

$$\zeta\left(\frac{1}{2}, \frac{1}{2} + x^2\right) \approx \begin{cases} -C_1 - C_2 x^2, & x \ll 1, \\ -2x - 1/48x^3, & x \gg 1, \end{cases} \quad (12)$$

$$C_1 = (1-\sqrt{2})\zeta(1/2) \approx 0.605, \quad C_2 = (1/2-\sqrt{2})\zeta(3/2) \approx 2.39. \quad (13)$$

With the help of this expansion, we establish two limiting cases of Supplementary Equation 11. The limit of negligible spin-orbit scattering $l_{\mathrm{SO}}^{-1} = 0$ corresponds to a metal with orthogonal symmetry exhibiting weak localization,

$$\Delta\sigma^{\mathrm{orth}}(B) = \frac{e^2}{2\pi h l_B}\left[\zeta\left(\frac{1}{2}, \frac{1}{2} + \frac{l_B^2}{l^2}\right) - \zeta\left(\frac{1}{2}, \frac{1}{2} + \frac{l_B^2}{l_\phi^2}\right)\right].$$

This has been discussed in Supplementary References 2 and 3. The limit of extremely strong spin-orbit coupling $l_{\mathrm{SO}}^{-1} = \infty$ corresponds to symplectic symmetry and pure weak antilocalization. It differs only by a (negative) factor from the orthogonal case,

$$\Delta\sigma^{\mathrm{sympl}}(B) = -\frac{1}{2}\,\Delta\sigma^{\mathrm{orth}}(B). \quad (14)$$

This result (in a different form) was used to describe quantum interference in Weyl semimetals [4].

When fitting experimental data, we consider lowest magnetic fields where $l_B \gg l, l_{\mathrm{SO}}$. In this case, only the second zeta function in Supplementary Equation 11 provides magnetic field dependence:

$$\Delta\sigma(B) = \mathrm{const} + \frac{e^2}{4\pi h l_B}\zeta\left(\frac{1}{2}, \frac{1}{2} + \frac{l_B^2}{l_\phi^2}\right). \quad (15)$$

Hence low-field data is fitted by the function with only two independent parameters: number of channels N, and dephasing length l_ϕ. For moderate values of magnetic field, such that $l, l_{\mathrm{SO}} \ll l_B \ll l_\phi$, expression (15) simplifies further and looses any dependence on material parameters:

$$\Delta\sigma(B) = \mathrm{const} - \frac{e^2 C_1}{4\pi h l_B}. \quad (16)$$

Supplementary Note 2. Details of the fitting procedure

Here, we present details of the fitting using Eq. 2 (low-field formula) in the main text.

1. Pick a magnetic field range for the fitting for the magnetoconductance curves, such that l_B is approximately ten times larger than l (as estimated from transport and band parameters).

2. Fit to determine N and l_ϕ at every temperature.

3. Report N for each sample as an average over a few lowest temperatures, with some standard deviation.

4. Using the average of N determined for the lowest few temperatures, redo the fits and extract l_ϕ versus T for fixed N. (To determine the temperature dependence of l_ϕ, we note that N should physically be a T-independent quantity.)

5. Additional fits were tried up to $\pm 30\%$ of the original magnetic field range to obtain an error due to the range of magnetic field used in the fitting.

In a few cases, using the average value of N caused a larger change in l_ϕ than the error obtained from changing field range, so this was accounted for in the error of l_ϕ. Thus, the error in N includes considerations from temperature averaging and range of magnetic field used, while the error in l_ϕ includes contributions from errors in N and also the range of magnetic field used.

Supplementary Note 3. Analysis of quasi-2D films based on 3D WAL formula

In the main text, quasi-2D films (d=50 nm) were analyzed using the 2D HLN formula[1]. We show an analysis based on the 3D WAL formula (Eq. 2 in the main text) in Supplementary Figure 2. The fit based on the 3D formula gives N values significantly larger than those obtained for 3D films with comparable n, signalling that the 3D WAL may not apply for these quasi-2D films.

Supplementary References

[1] S. Hikami, A. I. Larkin, and Y. Nagaoka, Prog. Theor. Phys. **63**, 707 (1980).
[2] A. Kawabata, Solid State Commun. **34**, 431 (1980).
[3] A. Kawabata, J. Phys. Soc. Jpn **49**, 628 (1980).
[4] H.Z. Lu and S. Q. Shen, Phys. Rev. B **92**, 035203 (2015).
